# Healthcare planning across healthcare sectors in Baden-Wuerttemberg, Germany: a stakeholder online survey to identify indicators

**DOI:** 10.1186/s12913-021-06514-0

**Published:** 2021-05-27

**Authors:** Pamela Wronski, Jan Koetsenruijter, Dominik Ose, Jan Paulus, Joachim Szecsenyi, Michel Wensing

**Affiliations:** 1 Department of General Practice & Health Services Research, Heidelberg University Hospital, Im Neuenheimer Feld 130.3, 69120 Heidelberg, Germany; 2 Present address: Department of Family and Preventive Medicine, University of Utah School of Medicine, 375 Chipeta Way, Salt Lake City, UT 84108 USA

**Keywords:** Health services administration, Cross-sectoral healthcare, Community health, Delivery of health care, Germany

## Abstract

**Background:**

Stakeholders in the German state of Baden-Wuerttemberg agreed upon the central aims for healthcare planning. These include a focus on geographical districts; a comprehensive, cross-sectoral perspective on healthcare needs and services; and use of regional data for healthcare planning. Therefore, healthcare data at district level is needed. Nevertheless, decision makers face the challenge to make a selection from numerous indicators and frameworks, which all have limitations or do not well apply to the targeted setting. The aim of this study was to identify district level indicators to be used in Baden-Wuerttemberg for the purpose of cross-sectoral and needs-based healthcare planning involving stakeholders of the health system.

**Methods:**

A conceptual framework for indicators was developed. A structured search for indicators identified 374 potential indicators in indicator sets of German and international institutions and agencies (*n* = 211), clinical practice guidelines (*n* = 50), data bases (*n* = 35), indicator databases (*n* = 25), published literature (*n =* 35), and other sources (*n* = 18). These indicators were categorised according to the developed framework dimensions. In an online survey, institutions of various stakeholders were invited to assess the relevance of these indicators from December 2016 until January 2017. Indicators were selected in terms of a median value of the assessed relevance.

**Results:**

22 institutions selected 212 indicators for the five dimensions non-medical determinants of health (20 indicators), health status (25), utilisation of the health system (34), health system performance (87), and healthcare provision (46).

**Conclusions:**

Stakeholders assessed a large number of indicators as relevant for use in healthcare planning on district level.

**Trial registration:**

Not applicable.

**Supplementary Information:**

The online version contains supplementary material available at 10.1186/s12913-021-06514-0.

## Background

German healthcare planners with responsibility for healthcare service provision are challenged by changing environmental factors such as increasing numbers of people living with chronic disease driven by the ageing of the population [1, 2] and shifts in morbidity [3]. This is further exacerbated by workforce shortages across the health professions which poses challenges for German health care planners in rural regions [4, 5]. A structural challenge within the German health system is the fragmentation of medical care across sectors [6]. In particular, capacity planning and budgeting for hospital and outpatient care are separate and a lack of integration with other service sectors such as health promotion and prevention, rehabilitation, mental healthcare, long-term care, pharmaceutical care, palliative care, social, and community also contributes to the current fragmented coordination. As a result, shortages as well as potential oversupply of healthcare services can be observed in communities and districts. The call for strengthening healthcare planning at local levels such as districts in Germany is further supported by study findings on regional variation in health status, equity, and healthcare delivery of the past decades [7, 8].

In the light of these issues, stakeholders, citizens, and patients in the state of Baden-Wuerttemberg (BW), a largely rural region with circa 11 million inhabitants, agreed on aims for improvements in healthcare services, the so called *Gesundheitsleitbild* (GLB). According to this, local actors should take responsibility for planning service provision and work on improving integration of healthcare between sectors, based on regional analyses of data on needs and services [9]. Against the background of the GLB the BW Ministry of Social Affairs and Integration (MSAI) initiated a model project to develop a concept for local and cross-sectoral healthcare planning supported by an information system delivering regional health system data.

There are many indicators and corresponding conceptual frameworks to provide information on health systems and their performance across countries [10], but they all have limitations or do not well apply to the targeted setting. Braithwaite et al. for example found in their comparative analysis across eight member countries of the Organisation for Economic Cooperation and Development (OECD) that only 45 out of 401 health system performance indicators were used in more than one of the compared countries [10]. Similar to the Canadian discussion on health reporting systems, a sort of “*indicator chaos*” [11] can be observed, primarily driven by sector specific aims and data providers, but also driven by the lack of a common indicator framework. Thus, local decision makers or potential data users, such as population health authorities on district level or local initiatives between healthcare providers, users and, payers, face the challenge to make a selection of indicators.

Furthermore, involving potential data users in indicator selection is expected to facilitate their awareness and use of results of analyses [12].

In this study, we aimed to identify relevant indicators for healthcare planning across sectors in the German state of BW, involving key stakeholders based on a developed framework for health system indicators.

## Methods

This study is part of *Subproject 1*, which aimed at the development of an indicator database to provide information on all districts in Baden-Wuerttemberg to support needs-based healthcare planning at district level. *Subproject 1* was part of a larger programme, the Model Project Cross-Sectoral Healthcare [13], which was initiated by the Ministry of Social Affairs and Integration of BW to explore how regional healthcare planning according the aims of the GLB could be conducted. For the efficient development of an indicator database, the overall project focussed largely on eight primarily chronic diseases: Anorexia nervosa, chronic lower back pain, colorectal cancer, dementia, depression, type 1 and type 2 diabetes mellitus, and stroke. These diseases were identified through a structured selection process following some criteria. These were mainly derived from the programme’s overall goals, i.e. there should be involved several sectors in the healthcare process of concerned patients, diseases should cover a broad spectrum (e.g. cardiovascular diseases, mental health), have a high prevalence in BW, be of relevance in different phases of life, the diseases should be influenceable through measures of prevention and health promotion, and data should be available. These considerations led to the following four selection criteria: there exist evidence-based guidelines (S3 according to the German classification of guidelines) [14], the disease has a high relevance for the morbidity of the population in BW, there exist data on morbidity from various sources, which are available on district level. The selection process is described more detailed elsewhere [15]. We decided to approximate health need, a broad concept which can be estimated in many ways, primarily by disease specific morbidity [16]. The time span of the project was from January 2016 until April 2018.

### Study design

We conducted a stakeholder online survey in style of a Delphi study’s first round. Generally, the Delphi method supports decision making in situations with either insufficient or excessive information by asking many experts at a time, usually via a postal or online questionnaire, if required with meetings [17, 18]. In this study, institutions from key stakeholder groups in the state’s healthcare system were asked to assess the relevance and comprehensibility of systematically investigated indicators through a standardised online questionnaire. The methodological approach was derived from the RAND/UCLA appropriateness method (RAM) [19] which is regularly used for the selection and development of healthcare quality indicators and also applied in German quality indicator development and healthcare planning in particular healthcare sectors. Especially the Delphi element of this method seemed to fit the study’s initial situation of making a choice from numerous available indicators. Participants of the online survey were informed in written form about the study context, the procedure of data collection, and data security. Participation was voluntary and only possible, when participants gave their informed consent to this. Data was collected and analysed anonymously on individual level. The research ethics committee of Heidelberg University Hospital waived ethics approval for the study.

**Fig. 1:**
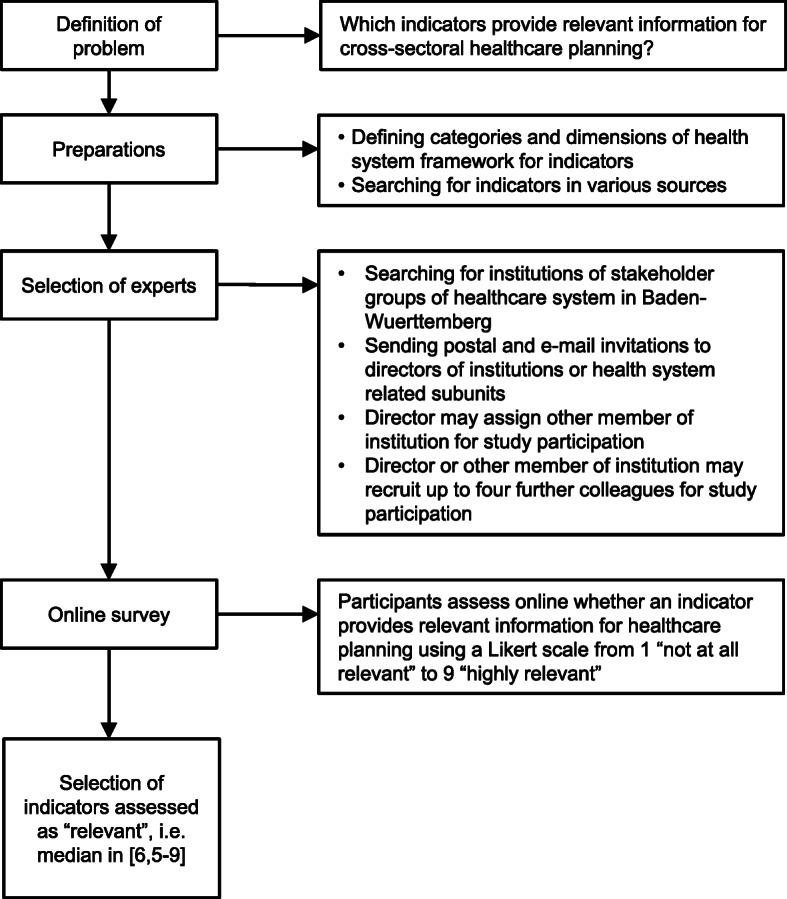
Process of indicator selection

The stakeholder survey was proceeded by the development of a conceptual framework for indicators and a structured search for indicators. The whole procedure from problem definition to indicator selection is outlined in Fig. 1.

### Conceptual framework and its development process

In order to guide the search and selection of indicators, we developed a conceptual framework together with a project group consisting of representatives from eight institutions, which were the MSAI, one population health organisation on state and three on district level, academic colleagues from two universities, and the authors. At the end, the framework aimed to be comprehensive, i.e. taking into account all areas of the health system (e.g. medical care in hospitals as well as primary prevention and health promotion), descriptive, i.e. mainly listing health system dimensions and arranging them in a hierarchical structure, and fit the project’s aims stated earlier.

First, the study group worked out a first version of the framework. The starting point was an international preselection of eight existing health (care) system frameworks listed by Arah et al. [20]. We regarded this preselection as sufficient, because it offered many dimensions arranged in various ways. The resulting first version of the framework was mainly derived from the Canadian Health Indicators Framework [21]. This seemed to fit best the aim of finding comprehensive and mainly descriptively arranged dimensions. The Canadian framework classifies not only health system performance indicators, but also indicators of public health. Its purpose is a comprehensive description of the health system. This coincided with the project’s goal to provide data on healthcare need and the corresponding supply. Furthermore, the programme was based on a broad concept of cross-sectoral healthcare, also comprising primary prevention and health promotion. Therefore, data on non-medical determinants of health, as covered by the Canadian framework were required as well. This first framework version comprising of four dimensions then was developed further on the grounds of feedback in project meetings and individual feedback from project group members, and resulted in the final version the project group agreed on.

The final version of the framework and the definition of its subdimensions are displayed in Table 1.

**Table 1:** Structure and definitions for sub-dimensions of the framework for indicators of the health system in Baden-Wuerttemberg

Framework sub-dimension	Description
Non-medical determinants of health	
Health behaviours	This sub-category includes self-harming and positive health behaviours. Actions of healthcare planning may aim to promote positive health behaviours.
Social determinants	Social determinants of health embrace the two sub-categories living and working conditions and environmental factors of the Canadian framework. According to the WHO, social determinants of health describe conditions individuals are born, grow up, live, work, and grow old with.
Demographic factors	Population characteristics such as age and gender fall under this sub-category.
Health status	
Morbidity	In this sub-category, primarily indicators concerning frequency of diseases focussed in the project are included.
Mortality	Information on mortality was to be collected mainly for the calculation of health system performance indicators but also to approximate regional health status.
Utilisation of the health system	
Prevention and health promotion	Through indicators assigned to this sub-category, utilisation of prevention or health promotion services and structures is measured.
Outpatient care	This sub-category includes the utilisation of services offered in practices, ambulatory healthcare centres, and domestic setting.
(Semi-residential) inpatient care	This sub-category subsumes the utilisation of services offered in hospitals, rehabilitation clinics, and nursing homes.
Health system performance	
Accessibility	Derived from the OECD’s Health Care Quality Indicators Framework accessibility defines how easy healthcare services are accessible. Access can be physical, financial, or psychological and requires the existence of the particular healthcare service.
Patient centeredness	Patient centeredness is achieved, when healthcare provision is orientated on patients’ wishes, expectations, and satisfaction.
Continuity	Continuity describes the degree to which healthcare provision for specific users is coordinated between health professionals and other institutions.
Effectiveness & efficiency	Effectiveness describes the degree to which a healthcare service achieves a desired result whereas efficiency means the optimal use of available resources to achieve maximum benefit.
Safety	Safety describes the degree to which healthcare processes avoid, prevent, or improve adverse events resulting from healthcare itself.
Healthcare provision	
Facilities	This sub-category includes a variety of health facilities with a focus of those which are especially relevant for patient groups selected in the project.
Professionals	Indicators of this sub-category were meant to include all health professionals having direct contact to either patients or their dependants such as physicians, psychologists, and nurses.
Technology	This sub-category subsumes health related products such as medical machines like computer tomography scanner, and telemedicine.
Honorary office	Besides health professionals health related support is also provided by other patients, e.g. in self-help groups or other patient organisations, and by other persons on a voluntary basis.

It differs from the first version as follows: Utilisation of the health system was added as fifth dimension. In the Canadian framework utilisation is not a separate dimension, but distributed over several other dimensions. Because the indicator set should enable analyses of cross-sectoral patient paths and an approximation of future demand of healthcare, we wanted the utilisation dimension and its indicators to be more visible in our framework. Other changes were additional sub-dimensions (social determinants, mortality) and separate sub-dimensions for professionals, technology, and honorary office. Looking at the arrangement of dimensions in the resulting framework from a health production process, health status is the centre. On the one hand this is influenced by non-medical determinants of health. On the other hand, it is influenced by health system factors, such as the utilisation of its services, by the way the health system performs, and the structure of health supply.

### Search for indicators

Based on the developed framework, a structured search for indicators was conducted by two of the authors. First, indicator sources were defined orientated on indicator source types described by the aQua-institute [22], a German research institute engaged in quality indicator development. Then, one of the authors selected indicators from the sources, if indicators matched at least one sub-dimension of the framework. Indicators with more than one possible sub-dimension were assigned to the final sub-dimension by another author. A total of 374 indicators from the following types of indicator sources were identified: indicator sets of German and international institutions and agencies (*n* = 211), clinical practice guidelines (*n* = 50), data bases (*n* = 35), indicator databases (*n =* 25), published literature (*n =* 35), and other sources (*n* = 18) such as a request of indicators from an academic project partner of the three district level public health authorities of the project group, and suggestions from the study team not found in a specific indicator source. A detailed list of indicator sources is provided in Additional file 1.

### Recruitment of stakeholders

The institutions who would be approached to assess the indicator set were to be stakeholders in the health system in BW. Since the range of indicator aspects was broad, we aimed to engage stakeholders being familiar with at least one dimension of the developed framework for indicators but not necessarily all dimensions. We identified five key stakeholder groups for our study, i.e. patients/citizens (PC), healthcare providers (HP), population health organisations (PHO), financing agencies (FA), and quality assurance agencies/statistical office (QAA/SO).

The sampling strategy was purposive sampling of institutions [23]. Some institutions, all from PHO, were already part of the project group. The group of PC consisted of representatives from self-help groups and other institutions providing patient support on a voluntary basis addressing patients with one of the focussed diseases. The stakeholder list included institutions specialised in all eight diseases or fields comprising these diseases. Furthermore, institutions representing citizens engaged in the living environment of other vulnerable groups were approached with the idea, that their expertise would be of value for especially indicators of non-medical determinants of health. The group of FA contained the largest - in terms of members - social and private health insurers, social pension schemes, and social accident insurances operating in BW. HP were listed through their representing organisations. We listed organisations of different health professions mainly relevant for providing healthcare to patients with one of the eight diseases, e.g. physicians, psychologists, nurses, physiotherapists, and occupational therapists. QAA/SO also included disease specific registers.

In total, we invited 54 institutions: 13 PC, six PHO, ten FA, 21 HP, and four QAA/SO. All institutions were informed and invited to participate in our study via post and a 3 days delayed e-mail recruitment. Both, the postal and e-mail invitation included a description of the online survey. We addressed the directors of the invited institutions, in some cases we addressed directors of subunits if only these were connected to the health system. Since the number of indicators and different topics was high and therefore their assessment could take more than three hours for a single person, directors were given the option to nominate four further members of their institution to participate.

### Questionnaire

The online platform used for the stakeholder survey was programmed and managed at the research group’s department. There, study information on the main task i.e. to assess the relevance of proposed indicators to inform healthcare planning across sectors, was given explicitly. The number of representatives from each participating institution was asked to be indicated.

The assessment view of a single indicator included its identification number, the dimension and sub-dimension it is ought to operationalise, and its name which summarised the indicator’s content. This made the name of many indicators more extensive than in their original source. Filling many indicator names with more information was a compromise made due to the large number of indicators, wherefore we decided to forgo further specifications of indicators such as operationalisation. Since our main interest in stakeholder involvement was the reduction of collected indicators, more precisely a reduction of circa one third, stakeholders were asked to assess the relevance of each indicator on a Likert scale from 1 (not relevant at all) to 9 (highly relevant). An example of the assessment view is given in Additional file 2.

For the global criterion of ‘relevance’ there can be found several definitions. In the RAM process the equivalent concept of relevance is appropriateness, which alludes to benefits and harms a medical intervention may hold for patients [19]. Carinci et al. defined an indicator as relevant, when “[it] measures an aspect of quality with high clinical importance, a high burden of disease or high health care use […]” [24]. Since our focus was to select not only indicators for health system performance, but also for other health system dimensions such as non-medical determinants of health, relevance in this study refers to the ability of an indicator to measure an aspect perceived as important for comprehensive healthcare planning by stakeholders, similar to the first point of the definition provided by Carinci et al.

Another question was about the indicators’ comprehensibility (yes or no), which referred to an indicator’s name, in order to estimate whether stakeholders felt that they understood what aspect is intended to be measured by the proposed indicator. Results of this assessment were planned to be used after the selection of indicators in order to derive which indicator name formulation needed a revision. For this purpose, a ‘yes or no’ assessment seemed sufficient. At the end, comments could be made for each indicator.

Finally, participants were asked to sort their institution into one of the stakeholder groups and rank each group according to the relevance of the role they should have in healthcare planning on a scale from 1 (low) to 7 (high). The idea of this question was to build weights for each stakeholder group in order to weight relevance ratings of the institutions by their associated stakeholder group.

Due to the high number of indicators to be assessed, some arrangements in the question mode were made to keep the withdrawal rate as low and data quality as high as possible: participants could activate a filter, which would only include indicators relevant for one or more selected diseases focussed in the project, already assessed indicators by any participant of an institution could be filtered, and the assessment area could be left and joined again during the field phase keeping the information about processed indicators of previous sessions.

### Data analysis

Data collected through the online questionnaire was processed and analysed with IBM SPSS Statistics Version 24 and Microsoft Excel 2010. We analysed indicator assessments on institutional level and closing questions about stakeholder ratings on individual level.

Relevance was the only selection criterion for an indicator as the aim of this study was to identify relevant indicators for healthcare planning from a stakeholder perspective. Also in other contexts of indicator development, relevance is used as central selection criterion [22]. In style of RAM, we used the median of stakeholder assessments. An indicator was classified as ‘relevant‘, when its median score was in the range between 6.5 to 9, as ‘uncertain‘ with scores in the range from 4 to 6, and as ‘not relevant‘ with scores in the range from 1 to 3. The selection process from problem definition to selecting ‘relevant’ indicators is summarised in Fig. 1.

Comprehensibility was analysed only for ‘relevant’ indicators. When an indicator had at least one rating for being not comprehensive, we adjusted more metadata for the project’s final report, especially the indicator’s name and a short description.

The goal of analysing the content of comments was to receive hints for the measurement and name adjustment of ‘relevant’ indicators. Therefore, content of comments was analysed for ‘relevant’ indicators, where no operationalisation was given in the indicator’s source and for ‘relevant’ indicators rated as ‘not comprehensible’ by at least one institution.

For identifying an institution’s stakeholder group, we analysed the institutions’ pseudonyms, which also were sent to institutions in order to login to the online assessment area. The pseudonyms previously were sorted to the according stakeholder group.

## Results

### Participating stakeholders

The online platform for the assessment of indicators was open for stakeholders between December 12th 2016 and the end of January 2017. In total, 35 individuals from 22 (41%) of the invited institutions participated. Group specific response rates were 100% for population health organisations and quality assurance agencies/statistical office, 60% for financing agencies, 24% for healthcare providers, and 8% for patients/citizens. From eight institutions more than one person agreed on participation to the study. There were four institutions, where two persons agreed on participation, three institutions with three potential persons, and one institution with a total of four. Participant numbers by stakeholder groups and their share on total participants are presented in Table 2.

**Table 2:** Composition of participating stakeholders

Stakeholder group	individuals	institutions	
	N	N	% of all participating stakeholders
Patients/citizens	1	1	4.5
Healthcare providers	8	5	22.7
Financing agencies	8	6	27.3
Population health organisations	11	6	27.3
Quality assurance agencies/statistical office	7	4	18.2
Total	35	22	100.0

The average number of indicators a participant assessed for its relevance was around 269 within a range from 14 to all 374 proposed indicators, in both cases by one institution. On average an indicator’s relevance was assessed by 15.8 institutions. The number of relevance assessments by different institutions for an indicator ranged from seven to 22. On the level of stakeholder groups, relevance was rated for all indicators by PHO, FA, and HP, whereas the group of QAA/SO did not rate 27 indicators which all belong to the sub-dimension of effectiveness and efficiency. The group of PC rated 55 (15%) indicators in total, which belong to the dimensions non-medical determinants of health and health status.

Comprehensibility was assessed for an average number of around 270 indicators per participant ranging from 13 to 374 indicators, in both cases by one institution. Vice versa, an indicator’s comprehensibility was rated by an average number of 16 participating institutions. The minimum number of comprehensibility assessments per indicator was nine, the maximum was 22.

Around 57% of 35 individuals from 73% participating institutions answered the question, how relevant one of the five proposed stakeholder groups should be for healthcare planning tasks in BW. No participant of the PC group answered this question. Median values for stakeholder relevance in healthcare planning were similar for all stakeholder groups (range 5.0 to 6.0). Due to this similarity and the high number of missing values of the stakeholder rating we decided not to build weights based on these ratings and thus not to weight stakeholders’ assessments on indicator relevance differently.

### Selected indicators

**Fig. 2:**
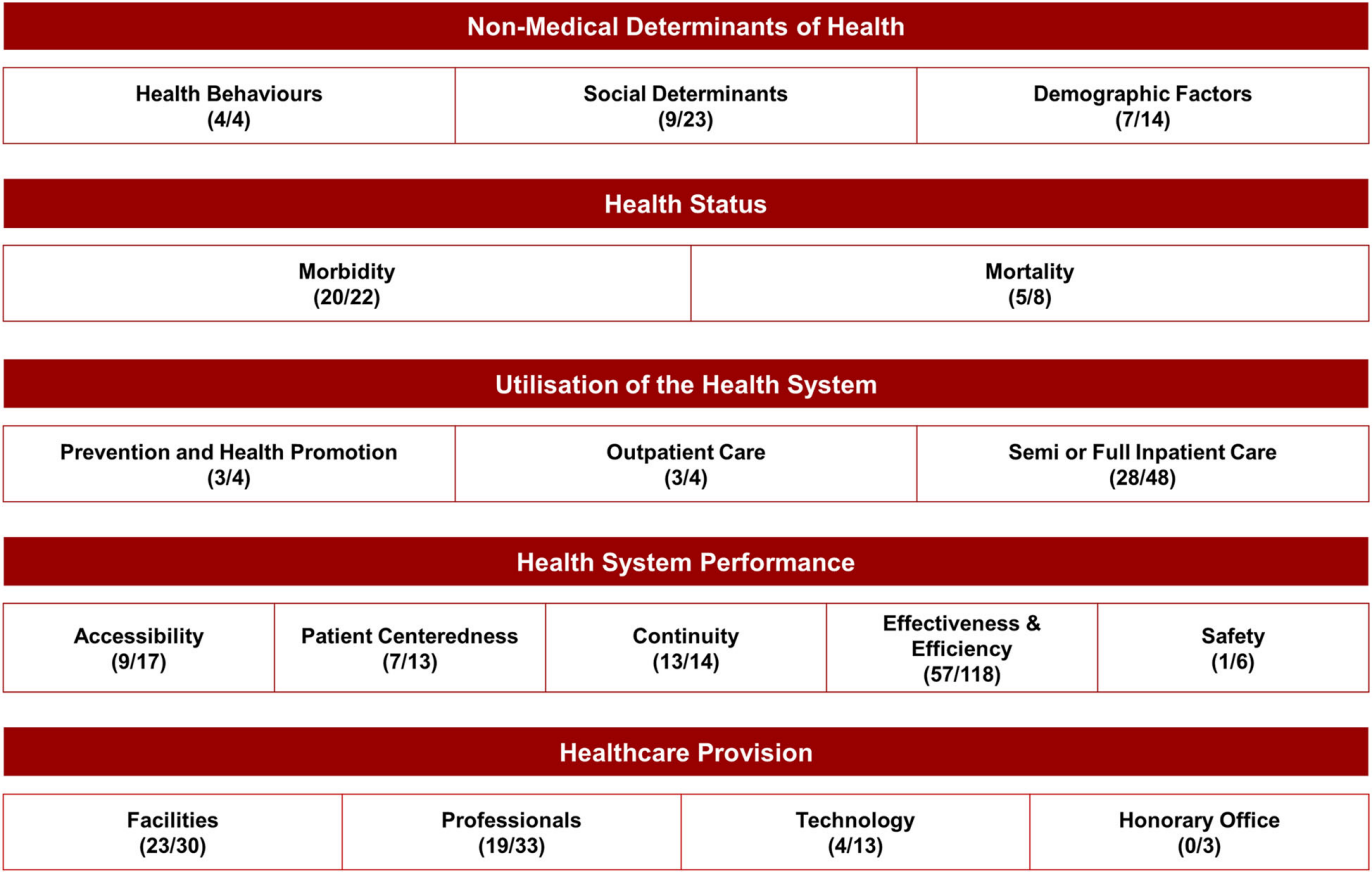
Framework for indicators of the health system in Baden-Wuerttemberg (number of selected indicators/number of proposed indicators)

From 374 proposed indicators 212 were classifiable as ‘relevant’ and therefore selected. 162 indicators were categorised either as ‘uncertain’ (*n* = 153) or ‘not relevant’ (*n* = 9) and were excluded from the final indicator set. Figure 2 shows the number of selected and proposed indicators for each framework sub-dimension.

The only indicator assessed with a median equal to nine (highly relevant) was physical activity. Most of selected indicators were related to the health system performance sub-dimension of effectivity and efficiency (27%) followed by semi or full inpatient care (13%) as sub-dimension of health system utilisation and facilities (11%) as sub-dimension of healthcare provision. More than 90% of proposed indicators of the sub-dimensions health behaviours, continuity, and morbidity were regarded as relevant whereas none of the three proposed indicators was selected from the sub-dimension of honorary office and less than a third of proposed indicators was selected of the sub-dimensions patient safety and technology.

Table 3 shows the institutions’ relevance ratings of indicators summarized for framework sub-dimensions. Due to the large number of indicators relevance assessments for each indicator is provided in Additional file 1.

**Table 3:** Relevance ratings of institutions (*n* = 22) by framework sub-dimensions ((mean/min/max) median per indicator)

Sub-dimension	All indicators (*n* = 374)				Mean selected^b^ (*n* = 212)	Mean not selected^b^ (*n* = 162)	Difference selected - not selected
	Mean	SD	Min	Max			
Non-medical determinants of health							
health behaviours	7.9	0.7	7.0	9.0	7.9	-^a^	–
social determinants	5.8	1.6	2.0	8.0	7.3	4.8	2.5
demographic factors	6.7	1.0	5.0	8.0	7.6	5.8	1.9
Health status							
morbidity	7.3	0.7	6.0	8.0	7.4	6.0	1.4
mortality	6.8	0.7	6.0	8.0	7.2	6.0	1.2
Utilisation of the health system							
prevention & health promotion	7.3	0.8	6.0	8.0	7.7	6.0	1.7
outpatient care	7.0	0.7	6.0	8.0	7.3	6.0	1.3
semi or full inpatient care	6.5	0.8	6.5	8.5	7.1	5.8	1.3
Health system performance							
accessibility	6.2	1.1	6.5	8.0	7.1	5.2	1.9
patient centeredness	6.1	1.2	3.5	7.5	7.0	5.0	2.0
continuity	7.4	0.6	6.0	8.0	7.5	6.0	1.5
effectiveness & efficiency	6.1	1.4	3.0	8.0	7.3	5.0	2.3
safety	6.0	1.0	5.0	8.0	8.0	5.6	2.4
Healthcare provision							
facilities	6.8	0.7	5.0	8.0	7.1	5.8	1.3
professionals	6.5	0.8	4.5	8.0	7.1	5.7	1.4
technology	5.7	0.9	4.0	7.0	6.8	5.3	1.5
honorary office	6.0	0.2	5.5	6.0	-^a^	5.8	–

The figures presented in this table are based on the median value per indicator resulting from the relevance assessment of participating institutions. E.g. for the sub-dimension *health behaviours* the mean value over the median value for the 4 indicators of this subdimension is 7.9

Likert-type scale for relevance ratings (1 = *not relevant at all* to 9 = *highly relevant*)

*SD* standard deviation; ^a^ Either all or none of the indicators were selected; ^b^ Formal consent about the selection i.e. relevance of an indicator was defined by a median in [6.5–9]

Health behaviours had the highest mean value (7.9) over its four indicators, safety and honorary office had the lowest (6.0). Together with minimum and maximum median values, the difference in mean values over the median of selected and not selected indicators approximates the consensus about indicators’ relevance. It varied most for social determinants (2.5) and varied least for health behaviours, where all indicators were selected, and honorary office, where no indicator was selected.

**Fig. 3:**
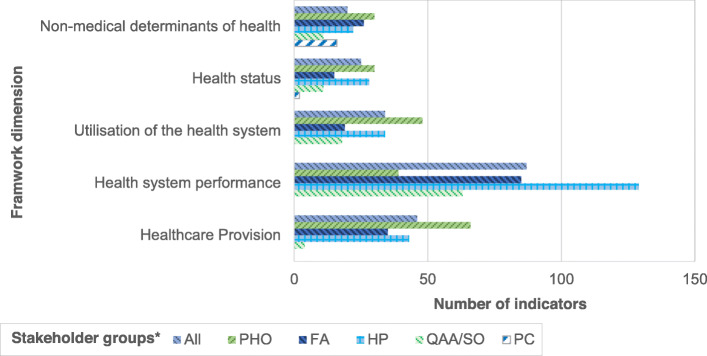
Number of relevant indicators by stakeholder group and framework dimension. * All: All stakeholder groups, PHO: population health organizations, FA: financing agencies. HP: healthcare providers, QAA/SO: quality assurance agencies/statistical office, PC: patients/citizens

Stakeholder specific relevance ratings resulted in different indicator selection throughout the five framework dimensions as displayed more detailed in Fig. 3.

The majority (90%) of ‘relevant’ indicators received not more than five comments. Two indicators were not commented and another two indicators had a maximum comment number of twelve. The content of all comments was interpreted as hint for indicator measurement and reformulation of an indicator’s name (e.g. notes to specify the indicator as well as questions about its unit).

## Discussion

To the best of our knowledge, for the first time in Germany, a comprehensive, multi-perspective stakeholder-based, set of 212 indicators for healthcare planning across healthcare sectors has been developed. This study shows that a stakeholder online survey can support indicator selection by different stakeholder groups of a health system. For the state of BW the indicator set can support the realisation of some stated key goals of the GLB, such as providing regional level data and considering healthcare need stronger in healthcare planning with indicators especially from the dimensions of health status and utilisation of the health system.

The aim of this study was to identify indicators to inform intersectoral and needs-based planning. The final indicator set provides intersectoral information in two ways: first, indicators of the subdimension continuity of care measure intersectoral aspects of care. Second, the indicator set is based on a comprehensive disease-specific approach: it delivers data on the healthcare needs for a patient group along different phases of disease, which are usually covered by various healthcare sectors. Further, the identified indicators provide data for needs-based healthcare planning. Analysing regional variation in need proxies such as morbidity and healthcare utilisation on the one hand and supply structures on the other could, e.g., help to identify regions where more action is needed to ensure sufficient healthcare. Additionally, these need proxies further can be projected on the basis of age and gender structures to approximate future need. This allows to estimate whether current supply structures will be sufficient in future.

There has been identified a large number of relevant indicators, but they are distributed over framework dimensions with a high variation from 57 indicators for effectiveness and efficiency to no relevant indicator at all in the sub-dimension of honorary office. This is mainly explained by the already variable number of indicators by sub-dimension given into the assessment. Thus, the indicator set does not deliver sufficient information on every framework sub-dimension. Although the indicator set was developed for the healthcare system in BW, all indicators could provide relevant information for other German states. Internationally, indicators of the dimensions non-medical determinants of health and health status could be most interesting for populations with similar morbidity structures. Hereby, it should be considered that morbidity indicators focus on eight specific and mainly chronic diseases. Indicators of other dimensions are mainly depending on health system structures and therefore might not be of relevance for other populations, when parts of health provision are organised differently.

Previously, indicator sets in Germany have been developed together with health system stakeholders or potential users in emergency response services [25]. A German state health ministry defined dimensions and sub-dimensions with a structure similar to a framework [26]. Nevertheless, we did not find indicator sets for cross-sectoral healthcare planning combining a predefined framework and indicator selection by different stakeholder groups through a formal selection process in Germany.

From an international perspective, there are practiced similar, more comprehensive approaches for selecting health system indicators as presented in this study, e.g. the OECD developed a conceptual framework and reviewed health care quality dimensions and indicators through a complete Delphi process [24]. In Italy, e.g. in the Tuscany region, indicators for regional healthcare planning were developed based on a framework and constantly enhanced involving different health system stakeholders in this process [27]. Beyond this, Italian regions integrated this information system in regional healthcare planning for public disclosure, as benchmarking tool, and basis for pay for performance governance [28].

The results of this study should be interpreted taking into account the following limitations. Firstly, the selected indicator set needs to be interpreted in the light of the study population’s composition. Our sample covers 35 individuals from 22 institutions and was selected purposively. This relatively small sample size and its sampling mode limit the representativeness of our study sample and its different groups. This means for example, that the study design and the small number of participants do not allow for deriving the general priorities or perception of the relevance of the indicators from stakeholder groups beyond the participants of this study. Instead, the results of the relevance rating can be regarded as a consolidation of a comprehensive body of expertise as there were participants across all the five defined stakeholder groups. We could not think of major or other institutions or stakeholder groups, and we felt that it was not feasible to recruit more individuals within the participating organisations and groups.

Response rates of stakeholders strongly differed by group ranging from circa 8% for PC to 100% for PHO and QAA/SO. This led to the smallest share for the group of PC among all participating stakeholder groups, which probably mostly explains that PC also rated the smallest portion of proposed indicators among participating groups. Studies on patient or citizen involvement in health policy making suggest, that patients’ or citizens’ priorities for health policy making can differ from those of professionals [29, 30]. Additionally, differences in indicator relevance ratings between the other stakeholder groups throughout all five dimensions support the suggestion, that selected indicators might have differed, if more PC related institutions would have participated in our study. There are many possible reasons for the especially low response rate among the stakeholder group of PC compared to the other groups. One reason could be associated with their honorary office in this position resulting in less time resources for additional tasks. Another reason might be that despite other stakeholder groups, patient or citizen organisations, e.g. self-help groups, are not conducting healthcare planning and therefore might not be familiar with related data and indicator concepts. As patients or citizens cannot be regarded as experts in the field of healthcare indicators for healthcare planning in spite of the other stakeholder groups, other methods for their inclusion might have been more appropriate [31, 32]. Future revisions of the indicator set should therefore consider to catch up on an additional indicator assessment by patient or citizen organisations considering more appropriate ways of including this perspective. The effect of other study population characteristics such as age and gender on the selection of indicators cannot be assessed in our study, because this data was not collected.

Secondly, the number of assessed indicators strongly varied among institutions. Some institutions started the assessment and stopped at a certain amount of indicators, which suggests that they did not finish the assessment. Also, more than half of the participating institutions assessed indicators through only one person and therewith did not make use of the option to distribute indicator assessment among other institutional members. Two participants reported that assessing all indicators took them more than three hours. The questionnaire length could have affected data quality in some cases [33] and might explain that some institutions did not finish the assessment. Other institutions skipped the assessment of mainly indicators from the subdimension of effectiveness and efficiency. These were partly very specific quality indicators, which required deep knowledge of the certain quality aspects addressed. This may indicate that participants preferably assessed indicators relevant or comprehensible to them. Though, we did not find any correlations between missing relevance ratings and low comprehensibility ratings. Also, nearly all indicators were assessed by all stakeholder groups but PC.

Furthermore, indicators referring to the concept of patient centeredness as defined in this study were planned to be included, however indicators found in the predefined sources did not reflect this concept sufficiently as none of them were patient-reported [34]. Nevertheless, patient-reported indicators for measuring patient centeredness exist [35]. This may indicate that the definition phase of possible indicator sources was dominated by the search for indicators with data available on district level and for all districts of BW. This, in turn, may indicate that a comprehensive measurement of patient reported indicators for health system performance evaluation is not yet institutionalised in BW and Germany. Currently, patient-reported indicators and their integration in nationwide quality assurance programmes are under development [36].

Finally, the conceptual framework was adopted from the Canadian health indicators framework, and has also adopted its limitations. A main concern is shown, when the indicator framework is applied: it does not provide information on how the dimensions and indicators relate to each other [37]. This might be further exacerbated by the high number of finally selected indicators. Orientation in the application of the indicators is provided by their relation to the eight diseases of the model project. The indicator search was orientated along these diseases so that the final indicator set provides information across the framework’s dimensions for each of the diseases. For practical implications we additionally checked the availability for each of the selected indicators. This showed that 19 indicators were directly available (publicly available online for free), 154 only after additional analysis on (non-)public data, and 39 indicators were currently not available. At the end of the project, data on indicators and their documentation were made accessible for free via the MSAI. Further development of the indicator set was not designed to be in the project. Most probable users of the indicator set may be PHO on state and district level. This stakeholder group already has a cross-sectoral perspective on healthcare planning on the one hand, but partly limited access to administrative health insurance data, which was one of the major data sources of the indicator set. In the last years, there have been made attempts to strengthen a local and cross-sectoral planning perspective in Germany. In BW a cross-sectoral board has been established in 2015 as defined by law [38]. Currently the task of this body is advisory. In this context the indicator set may be used to derive recommendations. On state and district level, there have been established committees called ‘health conferences’ in all districts in BW. On this platform local stakeholders of the health system can build cross-sectoral cooperation networks for specific topics. This could also function as a setting where the indicator set may be employed.

## Conclusions

The developed indicator set can support evidence-based decision making by regional healthcare planners in BW, who are responsible for healthcare planning on district and state level and strengthen a needs-based planning approach for regional healthcare structures. Specifically, PHO on state and district level may employ the indicator set. It may disclose and reduce potential gaps between data perceived as relevant and currently available. Also, it represents an approach how to select a large number of indicators including multiple perspectives in a formal way. With the new emergence of district level data, the indicator set should be updated. For healthcare planners in other countries, who consider developing or editing an indicator set for regional, cross-sectoral, and needs-based planning, our stakeholder online survey approach can provide a useful and efficient orientation.

## Supplementary Information

**Additional file 1.** List of proposed and selected indicators.

**Additional file 2.** Indicator assessment questions (example).

## Data Availability

The datasets analysed during the current study are available from the corresponding author on reasonable request.

## Abbreviations

BW: Baden-Wuerttemberg
GLB: Gesundheitsleitbild
MSAI: Ministry of Social Affairs and Integration
OECD: Organisation for Economic Cooperation and Development
RAM: RAND/UCLA appropriateness method
PC: Patients/citizens
HP: Healthcare providers
PHO: Population health organisations
FA: Financing agencies
QAA/SO: Quality assurance agencies/statistical office
