# Supplementary material for: Healthcare planning across healthcare sectors in Baden-Wuerttemberg, Germany: a stakeholder online survey to identify indicators
Source: BMC Health Serv Res. 2021 May 27;21:510. doi: 10.1186/s12913-021-06514-0 (PMC8157415; doi:10.1186/s12913-021-06514-0)

**Healthcare planning across healthcare sectors in Baden-Wuerttemberg, Germany: a** **stakeholder online survey to identify indicators**

Pamela Wronski^1^, Jan Koetsenruijter^1^, Dominik Ose^1,2^, Jan Paulus^1^, Joachim Szecsenyi^1^ and Michel Wensing^1^

^1^ Department of General Practice & Health Services Research, Heidelberg University Hospital, Im Neuenheimer Feld 130.3, 69120 Heidelberg, Germany. Email: [pamela.wronski@med.uni-heidelberg.de](mailto:pamela.wronski@med.uni-heidelberg.de), [jan.koetsenruijter@med.uni-heidelberg.de](mailto:jan.koetsenruijter@med.uni-heidelberg.de), [dominik.ose@hsc.utah.edu](mailto:dominik.ose@hsc.utah.edu), [janpaulus@gmx.net](mailto:janpaulus@gmx.net), [joachim.szecsenyi@med.uni-heidelberg.de](mailto:joachim.szecsenyi@med.uni-heidelberg.de), [michel.wensing@med.uni-heidelberg.de](mailto:michel.wensing@med.uni-heidelberg.de)

^2^ Present address: University of Utah School of Medicine, Department of Family and Preventive Medicine, 375 Chipeta Way, Salt Lake City, UT 84108, United States of America, Email: [dominik.ose@hsc.utah.edu](mailto:dominik.ose@hsc.utah.edu)

Corresponding author: Pamela Wronski, Department of General Practice & Health Services Research, Heidelberg University Hospital, Im Neuenheimer Feld 130.3, 69120 Heidelberg, Germany. Email: pamela.wronski@gmx.de

**Additional file 2. Indicator assessment questions (example)**


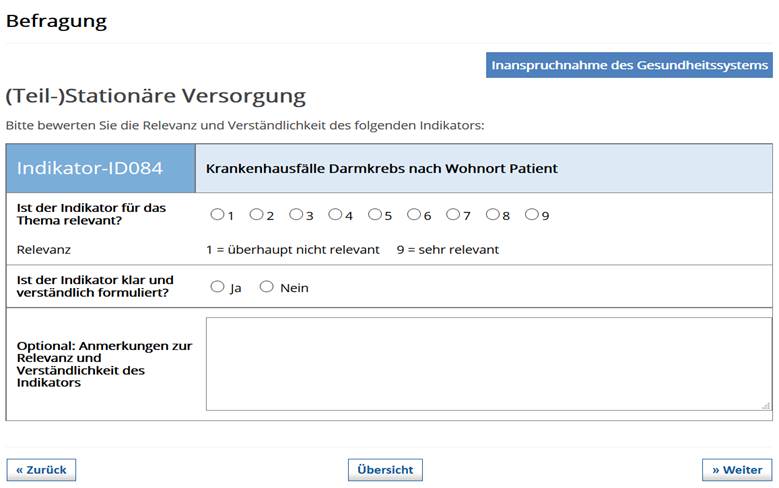

Supplement: Supplementary file 2 — Additional file 2. Indicator assessment questions (example). [file 12913_2021_6514_MOESM2_ESM.docx]
